# Control of protein stability by post-translational modifications

**DOI:** 10.1038/s41467-023-35795-8

**Published:** 2023-01-13

**Authors:** Ji Min Lee, Henrik M. Hammarén, Mikhail M. Savitski, Sung Hee Baek

**Affiliations:** 1 Graduate School of Medical Science & Engineering, Korea Advanced Institute of Science and Technology, Daejeon, 34141 Korea; 2 Genome Biology Unit, European Molecular Biology Laboratory, 69117 Heidelberg, Germany; 3 Creative Research Initiatives Center for Epigenetic Code and Diseases, School of Biological Sciences, Seoul National University, Seoul, 08826 Korea

**Keywords:** Biochemistry, Post-translational modifications

## Abstract

Post-translational modifications (PTMs) can occur on specific amino acids localized within regulatory domains of target proteins, which control a protein’s stability. These regions, called degrons, are often controlled by PTMs, which act as signals to expedite protein degradation (PTM-activated degrons) or to forestall degradation and stabilize a protein (PTM-inactivated degrons). We summarize current knowledge of the regulation of protein stability by various PTMs. We aim to display the variety and breadth of known mechanisms of regulation as well as highlight common themes in PTM-regulated degrons to enhance potential for identifying novel drug targets where druggable targets are currently lacking.

## Abstract (web summary)

Here the authors summarize current knowledge of the regulation of protein stability by various post-translational modifications (PTMs) including methylation and phosphorylation. PTM-regulated degrons act as signals for protein degradation or stabilization.

## Introduction

Out of the different biological macromolecules, proteins exhibit the most functional and structural variation. Most cellular and physiological processes, from metabolism and catalysis to signaling and locomotion, are enacted by proteins, making them essential players in homeostasis as well as in the development of diseases. While the foundation of the structural and functional diversity of a cell’s proteome lies in the gene-encoded primary polypeptide sequences of proteins, this basic framework is vastly enriched by alternative splicing of transcripts, as well as a wide array of post-translational alterations. The number of human protein isoforms generated by alternative splicing alone has been estimated to be around 100,000, pushing the total number of proteoforms generated by splicing and post-translational modifications (PTMs) to the tens of millions at least^1^. PTMs are covalent, enzymatic, or non-enzymatic attachments of specific chemical groups to amino acid side chains^2–5^. Even though the total number of known distinct PTM types has grown well beyond the 300 mark^4^, the most-studied non-proteinaceous PTMs remain enzyme-catalyzed phosphorylation, acetylation, methylation, glycosylation, and palmitoylation, as well as the nonenzymatic glycation and nitrosylation^6^. PTMs can also consist of separate polypeptides or protein domains conjugated via isopeptide bonds. In addition to classical ubiquitination and SUMOylation, modifications by other ubiquitin-like molecules (UBls) have increasingly also gained attention^7^.

PTMs can affect all aspects of protein function, one of which is a protein’s proteolytic stability. The primary PTM involved int the regulation of protein stability is ubiquitination, which operates through the ubiquitin-proteasomal system (UPS). The UPS is broadly implicated in diverse cellular pathways controlling, among other things, the activation of signaling cascades for differentiation, cell growth, and proliferation^8,9^. Ubiquitination generally occurs in a three-step, ATP-dependent process in which ubiquitin is first activated by an E1 enzyme, then conjugated to ubiquitin carrier E2 enzymes, and finally ligated to lysine residues of target proteins via specificity-conferring E3 ubiquitin ligase complexes^9^. A target lysine site can be modified by mono-ubiquitination or by poly-ubiquitination, by linking multiple ubiquitin molecules in a chain, where the next ubiquitin is added onto a lysine residue from the previous. Classical K48-linkage polyubiquitination (single-letter amino acid code denoting the amino acid position on ubiquitin) shuttles proteins for proteasomal degradation. As with other reversible PTMs, E3 ligases are counteracted by eraser enzymes, called deubiquitinases (DUBs). In addition, numerous non-proteolytic regulatory roles of mono- and poly-ubiquitination with other linkage modes have been described^10,11^. Ubiquitination is rarely the direct point of regulation, but rather preceded by the addition or removal of other PTM signals at or near the ubiquitination site. These PTMs act as recognition sites for reader proteins to interpret and integrate upstream signals providing an additional layer of rapid and reversible regulation and fine-tuning before committing a target protein irreversibly for degradation^12,13^.

A proteome-wide, mass spectrometry-based assessment of cross-talk between phosphorylation and ubiquitination in budding yeast revealed 466 proteins (constituting ~20% of all detected phosphoproteins) simultaneously carrying ubiquitination and phosphorylation^14^. Interestingly, around half of the ~2,000 phosphorylation sites identified on these doubly-modified proteins were exclusive to the ubiquitin-modified proteoforms. This subset constitutes prime candidates for having regulatory roles linked to ubiquitination, either upstream as ubiquitination-inducing phosphorylation, or as downstream effector modifications induced by ubiquitination. Indeed in this system, proteasome inhibition further indicated that phosphorylation sites likely to regulate ubiquitination and protein stability were in general closer to the ubiquitination site and more conserved evolutionarily than other phosphosites^14^.

In this review, we address the regulation of protein stability through PTMs with a focus on the emerging field of protein stability control by methylation. We aim to highlight different molecular mechanisms by which PTM-regulation is enacted and translated into changes in a protein’s proteolytic stability. These mechanisms range from the simple PTM-activated destruction signal (i.e. degron) to changes in a protein’s oligomericity or subcellular localization, which ultimately lead to altered protein degradation. Although we are focusing on the PTMs mainly responsible for controlling protein degradation or stabilization mediated by the UPS in this review, some of PTMs are involved in the regulation of protein stability through lysosomal and non-proteasomal degradation pathways.

PTM-control of protein stability is crucial for homeostasis and aversion to disease^15^. For instance, many proteins essential for cellular signal transduction are UPS targets^16^, and defects in their shuttling to the ubiquitination pathway can lead to disease by directly or indirectly influencing cell survival and proliferation^9,17^. Indeed, aberrant changes in protein turnover are among the dominant molecular characteristics of several diseases^18,19^, since a failure to recognize and consequently degrade proteins can eventually cause accumulation of unwanted signaling enzymes or even misfolded proteins^20,21^. Consequently, understanding the dynamics and spectrum of PTMs and exploring their functional significance in diseases could lead to the development of strategies for effective intervention, prevention, and therapy. Finally, we propose how new advances in quantitative proteomics could be used to systematically study and further elucidate PTM-driven protein stability control to rapidly expand the catalog of potential drug targets.

## Degrons as multicomponent control modules

**Fig. 1:**
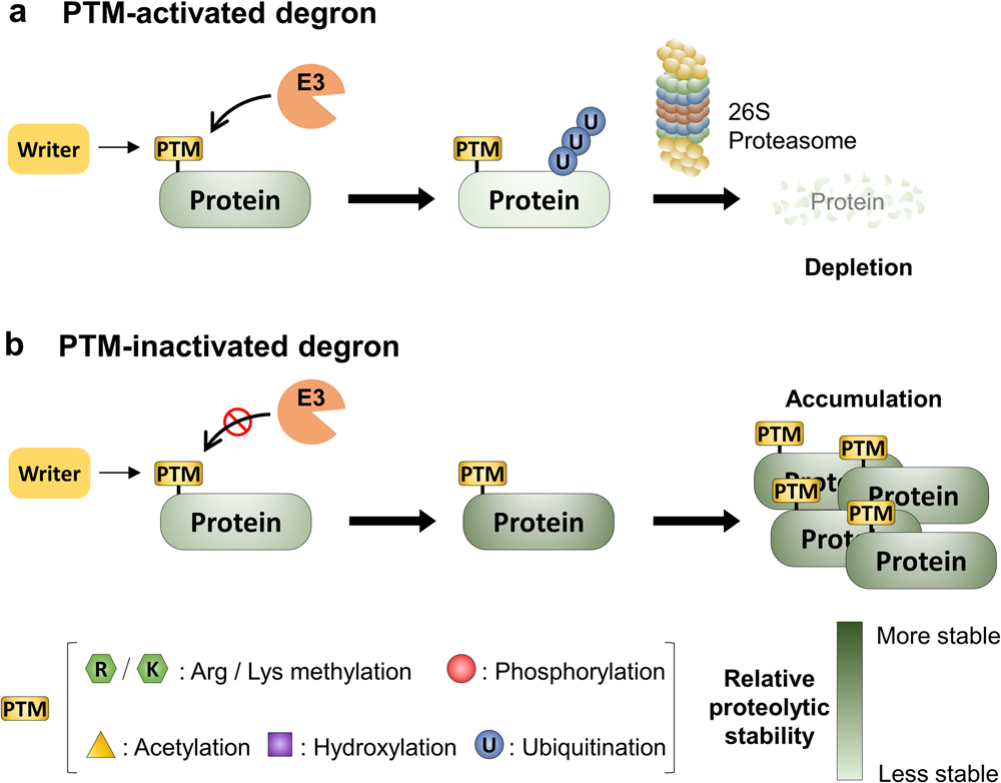
Examples of two opposite archetypal regulatory modules: the PTM-activated degron and PTM-inactivated degron. **a** PTM-activated degron: Writer enzymes generate PTMs at specific residues within target proteins that are recognized by an E3 ubiquitin ligase complex for proteasome degradation. PTM-activated degron is a natively inactive degron structure, modified, or otherwise activated by the addition of one or more PTMs, ultimately leading to the proteolytic degradation of a protein. **b** PTM-inactivated degron: PTMs added to specific sites on protein substrates to inhibit their interaction with the protein degradation machinery. PTM-inactivated degron is a destabilization motif that is inactivated by the addition of one or more PTMs.

Degrons were originally defined as protein motifs causing “metabolic instability of some or all of the peptide bonds in a protein”^22^. Since, degrons have been variously molecularly defined, specifically constituting the binding site of certain substrate-recognition subunits of E3 ligase complexes^23^. In this review, for PTM-driven regulation, we aim to highlight two main functional classes. First, the PTM-activated degron: a natively inactive degron structure, modified, or otherwise activated by the addition of one or more PTMs, ultimately leading to the proteolytic degradation of a protein (Fig. 1a). Second, the PTM-inactivated degron: a destabilization motif that is inactivated by the addition of one or more PTMs (Fig. 1b). It should be noted that the molecular mechanisms behind these classes are varied, and protein stability may be regulated by PTMs by indirect means, such as by providing binding sites for secondary proteins, which in turn prevent or indirectly induce degradation.

## Control of protein stability by methylation

Protein methylation was observed in the early 1960s, lysine methylation of histones a few years later, and arginine methylation of histones was subsequently discovered in the early 1970s^24,25^. Similar to other PTMs, two groups of writer and eraser enzymes, methyltransferases and demethylases, dynamically regulate the methylation state of target proteins^26^. Lysine residues offer three possible forms of methylation—mono-methyl, di-methyl, and tri-methyl–while for arginine residues, mono-methyl, symmetric di-methyl, and asymmetric di-methyl forms have been observed. Methylated residues act as binding sites for various reader domains, including MBT, Tudor, PWWP, chromo-, WD-40, ADD, ankyrin repeats, and PHD finger domains^27^.

### Methyl-activated degrons

**Fig. 2:**
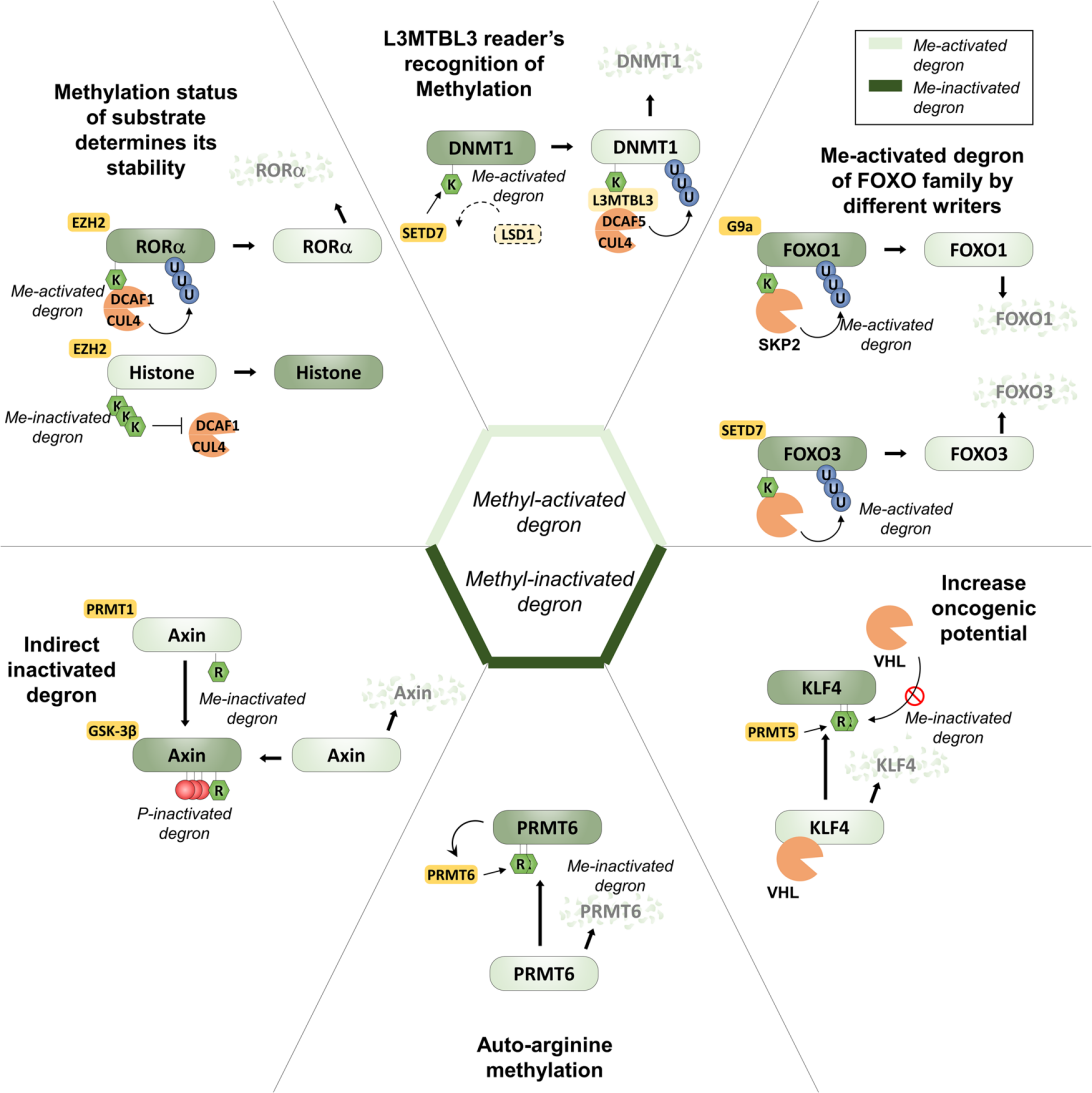
Molecular function of lysine and arginine methylation in control of protein stability. Lysine and arginine methylation regulates the function of protein by altering stability. Of note, lysine methylation converts protein stability negatively by methyl-degron (light green) and arginine methylation mainly increases protein stabilization by methyl-inactivated degron (dark green). Methyltransferase EZH2, G9a, and SETD7 modify lysine site via mono-methylation and further stimulate poly-ubiquitination of substrates by reader protein such as DCAF1 and L3MBTL3.

Several apparent methyl-activated degrons have been described^28,29^ (Fig. 2). Retinoid acid-related orphan receptor α (RORα)^30^, whose stability has been shown to be regulated by a lysine methyl-activated degron during oncogenesis, is one such example. RORα is mono-methylated at K38 by the enhancer of zeste homolog 2 (EZH2) methyltransferase. When methylated, this residue is recognized by the reader protein DCAF1 (damage-specific DNA-binding protein 1 (DDB1)-cullin4 (CUL4)-associated factor), leading to the recruitment of DDB1/CUL4 E3 ubiquitin ligase complex and poly-ubiquitination of RORα. In contrast to the histone substrates of EZH2, which are di- or tri-methylated, RORα is mono-methylated by EZH2 and then specifically recognized by DCAF1 with its putative chromodomain linking mono-methylation to protein degradation^30^. Interestingly, EZH2 is frequently mutated or overexpressed in cancers, and its functions have been characterized mainly as a master transcriptional regulator in its role as the active histone methyltransferase subunit of the Polycomb repressor complex (PRC)^29^. Findings of an inverse correlation between EZH2 and RORα protein levels in breast cancers, where protein levels of EZH2 are increased and RORα reduced^30^, suggest that part of the oncogenic role of EZH2 could also be through facilitation of the degradation of RORα described above since RORα is a known tumor suppressor in breast cancer.

Among various lysine methyltransferases that modify non-histone proteins as well as histones, SET domain containing 7 (SETD7, also called SET7, SET9, SET7/9, or KMT7) is one of the most extensively studied ones, given that most of the non-histone proteins possessing methyl-activated degrons have been identified as SETD7 substrates. SETD7 is responsible for methylation of nuclear factor (NF)-κB subunit RELA (also-called p65) at K314 and K315 upon tumor necrosis factor-α (TNF-α) stimulation^31^. This SETD7-dependent methylation of RELA is a prerequisite for RELA protein degradation and following attenuation of transcription of NF-κB target genes. Interestingly, this degron is further controlled by acetylation of the nearby K310 in an example of acetylation-methylation crosstalk. Acetylation of RELA K310 by the acetyltransferase p300 increases the stability and transcriptional activity of RELA by suppressing SETD7-mediated methylation probably by interfering with the interaction of SETD7 and RELA^31^.

Hypoxia-inducible factor α (HIF-1α), one of the most iconic proteins controlled by degradation, was also identified to be a SETD7 substrate and potentially carry two SETD7-dependent methyl-activated degrons: K32 and K391, which are constitutively methylated in normoxic conditions^32^. Both sites were demethylated by lysine-specific demethylase 1 (LSD1, also KDM1A), which prevented HIF-1α poly-ubiquitination and degradation by the UPS, and thus increased HIF-1α target gene expression^32^. Notably, from a clinical perspective, LSD1 overexpression could induce HIF-1α target gene expression even in normoxic conditions. Mechanistically, K391 methylation of HIF-1α was shown to be dependent on the oxygen-sensing hydroxylation on P402 and P564, and LSD1 was suggested to attenuate both of these modifications through increased LSD1 expression and activity upon hypoxia, although the detailed molecular mechanism for this is unclear.

Another SETD7 target is DNA methyltransferase 1 (DNMT1)^33^, which itself methylates cytosines in CpG dinucleotides, and whose activity is tightly regulated during cell cycle progression and is vital for cancer progression^34^. Several groups have shown that the mono-methylation of DNMT1 by SETD7 at K142 triggers DNMT1 protein degradation^33,35^. In fact, K142 is part of a consensus methylation motif on DNMT1, which can also be found on E2F1, a critical transcription factor for cell cycle regulation. Like on DNMT1, E2F1 K185 methylation by SETD7 causes targeting of the E2F1 protein to the UPS^36^. In both cases, the methylated degron motifs are recognized by an MBT domain-containing protein L3MBTL3, which recruits the CUL4/DCAF5 E3 ligase leading to degradation by the UPS^35^. Similarly to HIF-1α, the action of SETD7 is counteracted by the demethylase LSD1.

Another example is Sterol regulatory element-binding protein 1 (SREBP1/SREBF), which abnormally activates lipogenesis to high levels in cancer cells and promotes growth. SREBP1 is specifically di-methylated at R321 by protein methyl arginine transferase 5 (PRMT5), thus stabilizing SREBP1 via a reduction in GSK-3-mediated phosphorylation of S430–a site previously implied as a phospho-degron recognized by the substrate recognition subunit FBXW7 of the SKP1–cullin1 (CUL1)–F-box protein (SCF) E3 ligase (or SCF^FBXW7^ for short) (Ref. ^37^). However, the molecular mechanism of pS430 reduction is yet to be discovered.

The forkhead transcription factor (FOXO) family members are regulated by numerous PTMs, including methylation. Recent evidence indicates that FOXO1 methylation by the methyltransferase G9a (or euchromatic histone lysine methyltransferase 2, EHMT2) at K273 leads to increased interaction between FOXO1 and the E3 ligase S-Phase kinase-associated protein 2 (SKP2), resulting in decreased FOXO1 protein stability by accelerating poly-ubiquitination and degradation of FOXO1 (Ref. ^38^). Similarly, for FOXO3, methylation at K271 by SETD7 also results in decreased FOXO3 protein stability via the UPS. In both these cases, the methylation is relatively close to the known indirect phospho-degron at S256 and S253, respectively, but the mechanism of action has not been further elucidated. FOXO1 and G9a have a potential pathological and clinical relevance, as it has been observed that FOXO1 protein levels are reduced and G9a protein levels elevated in human colon cancer patient specimens^38^. Similarly to the case of an inverse correlation between oncogenic EZH2 and tumor-suppressive RORα in breast cancer, we speculate that the potential oncogenic function of G9a might be augmented by the degradation of FOXO1, which is a known tumor suppressor.

Coactivator-associated arginine methyltransferase 1 (CARM1, also known as PRMT4) has been shown to be essential for estrogen-induced cell cycle progression in breast cancer^39^. Upon estrogen stimulation, ERα is recruited to the target promoters along with increased methylation of histone H3 arginine 17 by CARM1 for transcriptional activation of target genes. The recruitment of CARM1 to the ERα target promoters is dependent on nuclear receptor coactivator 3 (NCOA3; also known as steroid receptor coactivator-3, SRC-3), which is overexpressed in aggressive breast cancers together with CARM1^39^. NCOA3 has been shown to be methylated by CARM1, and mutagenesis and knock-down *in cellulo* experiments suggest that this methylation leads to a decrease in NCOA3 coactivator activity^40^, probably by decreasing NCOA3’s proteolytic stability^41^. The exact molecular mechanism also here, however, is yet to be elucidated.

Most known methyl-activated degrons rely on the UPS for protein degradation, and most methyl-activated degron-containing non-histone substrates identified thus far are transcription factors. It remains to be seen, whether these generalizations are historical biases introduced by the technical approaches traditionally used to identify such targets, or whether they will be corroborated by the advent of unbiased proteome-wide methods to detect methyl-activated degrons. Compared to lysine methyl-activated degrons, arginine methyl-activated degrons have not been explored as extensively^42^.

### Methyl-inactivated degrons

In contrast to the methyl-activated degrons described above, which directly or indirectly induce protein degradation upon the addition of a methyl group by recruiting E3 ligases, methyl-inactivated degrons either proteolytically destabilize their protein in their unmodified form or act as binding sites for stabilizing interactions when methylated (Fig. 2).

Therapeutically, one of the potentially most promising methyl-inactivated degrons has been described for kruppel like factor 4 (KLF4), which was initially identified as a tumor suppressor in many types of cancers, including gastrointestinal, esophageal, lung, and pancreatic cancer, but later found to be a mitogenic factor in breast cancer and squamous cell carcinoma^43^. Pull-down experiments of KLF4 identified PRMT5 as an interaction partner, which methylates KLF4 on three arginines (R374, R376, and R377) near its C-terminus^44^. This methylation was shown to decrease KLF4 poly-ubiquitination by the Von-Hippel-Lindau tumor suppressor (VHL) E3 ligase, increasing KLF4 stability, and abundance as well as transcription of KLF4-dependent target genes. Overexpression of wild-type KLF4 is tumorigenic, and methylation-incompetent mutated KLF4 was unable to initiate tumorigenesis in a breast cancer xenograft model, highlighting the importance of the methyl-inactivated degron for the oncogenic function of KLF4. More recently, efforts to produce small-molecule antagonists targeting the PRMT5-KLF4 interaction have further shown that pharmacological inhibition of the KLF4 methyl-inactivated degron is a potentially viable route in suppressing KLF4-dependent tumorigenesis. Another potential methyl-inactivated degron has been identified in the arginine methyltransferase PRMT6 itself, which auto-methylates itself at a conserved arginine residue at position 35 (Refs. ^45, 46^). Mutagenesis experiments showed that both PRMT6 catalytic activity and methylation at arginine 35 were critical for the proteolytic stability of PRMT6, thus suggesting the presence of a methyl-inactivated degron at this site^45^, even though the molecular mechanisms are as of yet unclear.

One of the mechanistically most thoroughly elucidated examples of methylation events that stabilize a protein is the methylation of FOXO1 at two arginine residues (R248 and R250 in mice; R251 and R253 in humans) by PRMT1 on its Akt consensus phosphorylation site (RXRXXS/T)^47^. This methylation modifies the Akt consensus site and thus prevents phosphorylation at the adjacent S256 in humans and S253 in mice, which when phosphorylated, causes translocation of FOXO1 to the cytoplasm, where it could be degraded^47,48^. Thus, methylation indirectly stabilizes nuclear FOXO1 through PTM crosstalk by preventing phosphorylation of its phospho-degron, which would otherwise cause translocation to the cytoplasm and consequent degradation of FOXO1.

Another, similarly indirect methyl-inactivated degron has been proposed in Axin, a scaffold protein in the β-catenin destruction complex, and thus the negative regulator of Wnt signaling. Experiments with murine Axin in cellular models suggest that R378 is a substrate of PRMT1, leading to increased interaction between Axin and glycogen synthase kinase 3-β (GSK-3β)^39^. This leads to a reduction of Axin ubiquitination, presumably by increasing Axin phosphorylation by GSK-3β, which prevents degradation of Axin by the UPS^49^. In addition to the potential arginine methyl-inactivated degrons above, a few putative examples of lysine methyl-inactivated degrons have been found. For example, estrogen receptor α (ERα), a ligand-dependent transcription factor, stimulates proliferation and inhibits apoptosis in various cancers. ERα is methylated at K302 by SETD7 (Ref. ^50^). SETD7-mediated methylation of ERα causes ERα stabilization and increases recruitment of ERα to its target gene promoters.

Lysine methyltransferases EZH2, SETD7, and G9a are predominant writer enzymes responsible for methyl-activated degrons and LSD1 is a matched counterpart eraser enzyme of SETD7 that drives erasing of methylation from methyl-activated degrons. In contrast, for methyl-inactivated degrons, lysine methyltransferase SETD7, arginine methyltransferases CARM1, PRMT1, PRMT5, and PRMT6 are responsible for increasing protein stability of target proteins by eliminating methylation from methyl-inactivated degrons.

There are erasers that can antagonize writer functions for generating PTM-degron marks. Among them, LSD1 is involved in erasing lysine methylation of target proteins generated by SETD7, however, LSD1 functions as a direct substrate of suppressor of variegation 3-9 homolog 2 (SUV39H2), which protects poly-ubiquitination of LSD1^51^. SUV39H2 drives tri-methylation of LSD1 and consequently, increased half-life of LSD1 triggers its binding to the corepressor for element-1-silencing transcription factor (CoREST) complex that, in turn, finally facilitates LSD1 tethering to the substrate proteins^52^. These studies imply a potential therapeutic strategy of combining inhibitors of eraser and writer enzymes managing methyl-regulated degrons for the treatment of diseases. In sum, a large number of non-histone substrates containing methyl-activated or -inactivated degrons with corresponding methyltransferases writer enzymes and demethylase eraser enzymes were identified (Table 1).

**Table 1:** List of methyl-activated and -inactivated degron with corresponding writer and eraser

Substrate	Activated/ inactivated Degron	Lys/ Arg	Site (Me)	Mono/di/tri	Methyl-transferase (Writer)	Demethylase (Eraser)	Site (Ub)
HIF1α	Activated	Lys	K32	me1	SETD7	LSD1	K532/K538/K547
DNMT1	Activated	Lys	K142	me1	SETD7	LSD1	C-terminus
E2F1	Activated	Lys	K185	me1	SETD7	LSD1	K161/K164
p65	Activated	Lys	K314/K315	me1	SETD7	-	K28/K62/K195
FOXO3	Activated	Lys	K271	me1	SET7	-	K242/K259/K290/K569
RORα	Activated	Lys	K38	me1	Ezh2	-	C-terminus
FOXO1	Activated	Lys	K273	me1	G9a	-	K245/K248
ERα	Inactivated	Lys	K302	me1	SETD7	-	K302
p53	Inactivated	Lys	K372	me1	SETD7	-	K370/K372/K373/K381/K382
PRMT6	Inactivated	Arg	R35	me1, 2	PRMT6	-	-
KLF4	Inactivated	Arg	R374/R376/R377	me1, 2	PRMT5	-	K43
SRC-3	Inactivated	Arg	R1171	me1, 2	CARM1	-	K723/K786
Axin	Inactivated	Arg	R378	me1, 2	PRMT1	-	K789/K821

## Control of protein stability by phosphorylation

The majority of currently known cases of PTM-regulated protein degradation constitute phosphorylation of serine, threonine, or tyrosine residues. Protein kinases and phosphatases are the responsible writer and eraser enzymes, respectively, catalyzing the transfer or removal of phosphate groups to their substrates, and their actions are involved in practically all key cellular processes, especially those implicated in cell-cell communication and coordination. CDKs in cell cycle regulation, GSK-3α/β in Wnt signaling, RTKs itself in RTK signaling, and Akt in neurodegenerative disease are the main writer enzymes responsible for phospho-activated or inactivated degrons. The coupling networks of these writer kinases and E3 ligase complexes provide opportunities for signal integration and tight control of each critical signaling pathway. Herein we focus on four biological systems where phosphorylation has a critical function in protein stability regulation, as illustrated with representative examples.

### Phospho-activated degrons in the cell cycle

Periodic proteolysis has been the focus of many studies on cell division in eukaryotes, due to its early discovery and evident importance in generating an irreversible step in the progression through each cell cycle stage^53^. Indeed, a recent proteome-wide study of protein dynamics showed that nearly 20% of the ~5,000 quantified proteins significantly changed in abundance along the cell cycle^54^. Progression through the cell cycle is essentially orchestrated by two main players: cyclin-dependent kinases (CDKs) and two E3 ligases: the anaphase-promoting complex (APC/C; also-called cyclosome) and the SCF complex^23^. Together with various other factors, these enact spatial and temporal rhythms in the phosphorylation states and abundances of a vast array of effector proteins. Classic examples of timed protein degradation during the cell cycle include regulators of CDKs, like the CDK-activating cyclins and CDK-inhibiting protein families (Ink4, Cip, and Kip (CDKN genes in humans)^55^.

Both the APC/C and SCF are multimeric protein complexes built around a core scaffolding protein—APC2 and CUL1, respectively—that recruits other subunits, which in turn confers substrate-recognizing and enzymatic activities. Especially the substrate-recognizing subunits—called activators in APC/C, and F-box proteins in SCF—have traditionally been thought to be crucial for substrate specificity of the complex, and, thus the regulation of the E3 ligase activity^23,56^. The exact mechanism of timed recognition and degradation of APC/C and SCF substrates is still an area of active investigation, however. Currently, for the APC/C, regulation is thought to be dictated by multiple means, such as the current abundance and composition of the APC/C, subcellular localization of the APC/Cs and substrates, the APC/C phosphorylation state, as well as competition between potential substrates with different relative binding affinities to the APC/C^23^.

**Fig. 3:**
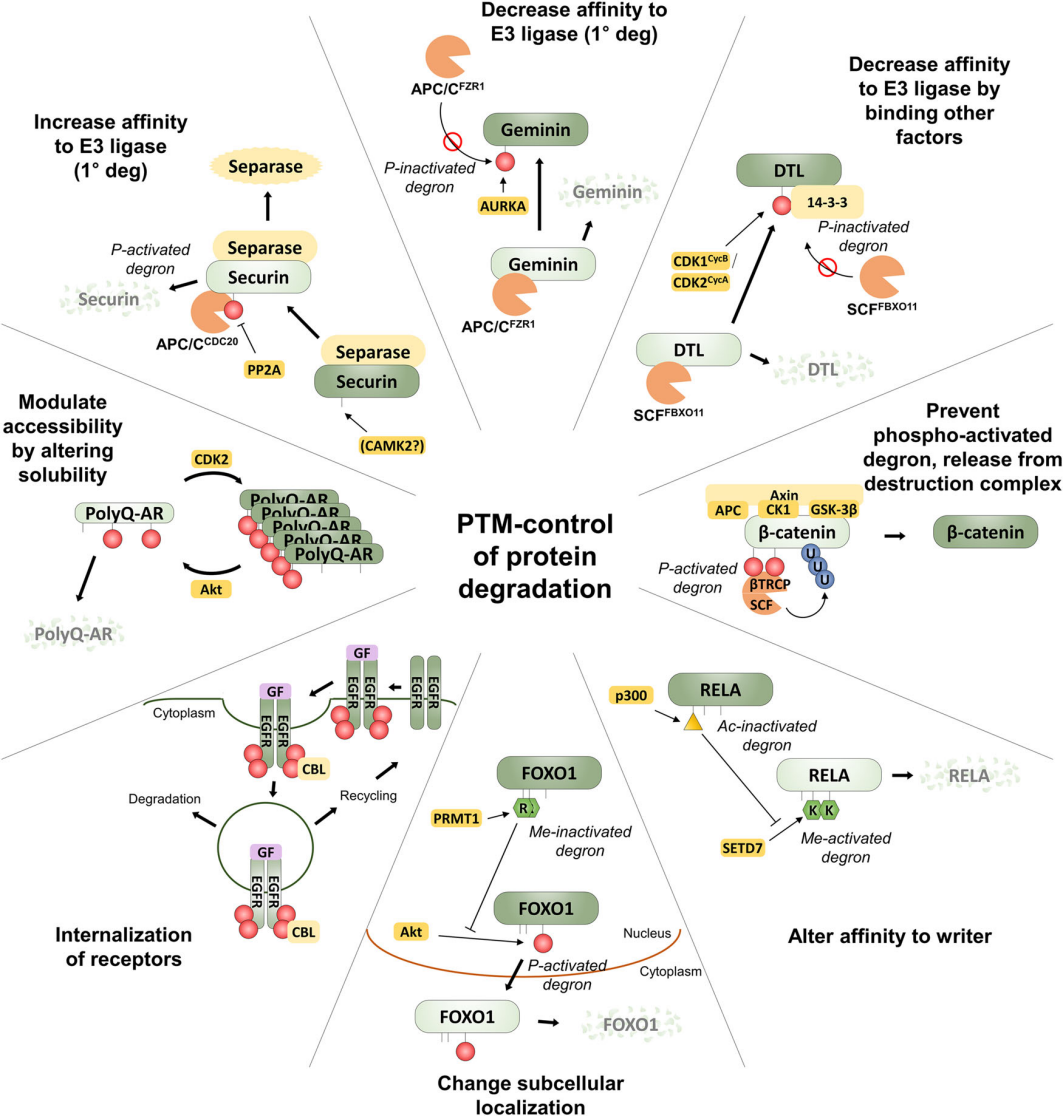
Diverse molecular mechanisms of PTM-control of protein degradation. Various different molecular mechanisms for PTMs changing a protein’s proteolytic stability have been found. DTL (for Denticleless protein homolog, also called CDT2) is the human orthologue of yeast Sic1 and itself a substrate adapter of the DCX E3 ligase complex. DTL is phosphorylated at T464, which promotes interaction with 14-3-3, thus shielding it from degradation by SCF^FBXO11^^44,167^.

PTMs can affect all of the above. An example of phosphorylation directly increasing affinity between the primary degron and its adapter is found in human securin, which is degraded more efficiently upon phosphorylation next to its D box degron motif^57^, as the introduction of the negatively charged phosphoryl group makes the motif more closely resemble the consensus D box motif sequence recognized by APC/C^CDC20^ (Ref. ^23^) (Fig. 3). In contrast, when the DNA replication inhibitor geminin is phosphorylated on its D box degron motif by Aurora kinase A^58^, an affinity for its adapter APC/C^FZR1^ is lost, thus effectively inactivating the degron (Fig. 3). Only when this phosphorylation is removed by phosphatases, can geminin be degraded during mitosis and the cell successfully enter a new cell cycle including DNA replication. Other notable APC/C substrates with reported phospho-inactivated degrons are cell division control protein 6 (CDC6) and SKP2, which itself is a substrate-recruiting F-box protein associated with the SCF E3 ligase^59–61^.

The regulation of substrate specificity of the SCF complex is even less well understood. Complicating understanding is the fact that there are 69 identified F-box proteins in humans, most of which are still orphans without known substrates^56^. Early research suggested that the SCF complex was specialized in binding phosphorylated substrates, and several examples of apparent phospho-degrons were found to be shuttled by the SCF complex to the UPS^56^. Previous reviews identified SCF complex adapter F-box proteins such as BTRC/FBXW1, FBXW11 (collectively called β-transducin repeat-containing protein, β-TrCP), as well as FBXW7 as strict phospho-degron-binding adaptors^56^.

Well-established examples of SCF-phospho-activated degrons, include Cyclin E, which is recognized and ubiquitinated by SCF^FBXW7^ only after phosphorylation at S384 by CDK2 and T380 by GSK-3, respectively^62,63^. Interestingly, biochemical and structural work on the Cyclin E phospho-activated degron, as well as another archetypal phospho-activated degron in the yeast Sic1 protein (recognized by the yeast SCF^Cdc4^ complex), also highlighted another important fact of substrate recognition by SCF (or indeed probably any E3 ligase)^64^. Namely, whether a protein is bound by an E3 ligase efficiently enough to allow for ubiquitination is determined not only by the binding affinity of a given degron to its E3 ligase substrate recognition subunit but also by the avidity of the entire interaction, which can be increased by dimerization or the inclusion of distal interaction regions^64^. This makes predictions of the effects of PTMs on degron activity fiendishly difficult. Cyclin E is also a prime example of the coexistence of multiple potential E3 ligase pathways a protein might be subjected to since Cyclin E has been shown to also be a potential substrate of the E3 ligase UHRF2 (also known as NIRF) in its unphosphorylated form, even though the physiological relevance of this is still unclear. Furthermore, Cyclin E also has an N-terminal, phosphorylation-independent degron recognized by CUL3, which is missing in some oncogenic Cyclin E mutants^65^.

A classical SCF substrate initially reported to be degraded in a phosphorylation-dependent manner is Cyclin D1. Cyclin D1 degradation has been linked to at least four different F-box proteins: SKP2 (Ref. ^66^), FBXW8 (Ref. ^67^), FBXW4 (Ref. ^68^), and FBXO31 (Ref. ^69^), out of which Cyclin D1 phosphorylated at T286 was shown to interact with and be ubiquitinated by SCF^FBXW4-alphaB crystallin^ and SCF^FBXW8^. While this superficially resembled a direct regulatory phospho-activated degron similar to the ones seen in other cyclins, it is mechanistically distinct, since Cyclin D1 T286 phosphorylation was later shown to promote interaction with a nuclear exportin, thus driving translocation of Cyclin D1 into the cytoplasm^70^, where it can subsequently be degraded by cytoplasmic SCF complexes, including SCF^FBXW8^ (Ref. ^67^). Furthermore, in addition to the cell cycle-dependent degradation described above, T286-phosphorylated Cyclin D1 was later shown to interact with SCF^FBXO31^ in response to DNA damage^69^. However, also in this instance, the relevance of phosphorylation for the interaction has been called into question, with recent structural and biochemical work showing that Cyclin D1 can be a SCF^FBXO31^ substrate in its unphosphorylated form in vitro^71^, highlighting the complexity of identifying and verifying the molecular mechanisms behind PTM-regulated degrons.

### The β-catenin phospho-activated degron

Canonical Wnt signaling is one of the key highly conserved signaling cascades for regulating development, stem cell proliferation, and cancer progression. As a major pathway controlling cell proliferation, aberrant activation of Wnt signaling also plays a critical role as a driving force in tumorigenesis^72,73^. At the heart of the Wnt signaling pathway, is a constitutive phospho-activated degron acting on the signal transducer β-catenin (CTNNB1) (Fig. 3). In the absence of stimulation, β-catenin is sequestered in the cytosol into the so-called destruction complex formed by adenomatous polyposis coli (APC), Axin, the protein kinases casein kinase I α/δ (CK1 α/δ, CSNK1A1/CSNK1D) and GSK-3α/β, as well as the SCF adapter β-TrCP. In this complex, β-catenin is successively phosphorylated at S45 by CSNK1 (ref. ^48^), and at T41, S37, and S33 by GSK-3. Together, phosphorylated S37 and S33 form the phospho-activated degron motif exemplified by DpSGXXpS, which is recognized by β-TrCP^74,75^, leading to ubiquitination and destruction of β-catenin, thus keeping free β-catenin levels low in the absence of stimulation.

Upon Wnt binding, the Wnt receptor–a heterodimer made up of Frizzled (FZD) and low-density lipoprotein receptor-related protein (LRP)–is activated and becomes able to recruit the β-catenin destruction complex via Axin and dishevelled (DVL)^76^. Following activation, the degradation of β-catenin is inhibited by a not fully understood mechanism^77^, and free β-catenin levels are allowed to increase, leading to the translocation of β-catenin to the nucleus and activation of downstream genes. Altogether, the β-catenin phospho-activated degron is one of the best-studied domains where phosphorylation and ubiquitination are dynamically regulated for the balance of positive and negative functions of β-catenin in Wnt signaling. It seems likely that phosphorylation has emerged as an upstream regulatory module for the regulation of ubiquitination and fine-tunes the pulsatile signaling and downstream transcriptional activation function of β-catenin in response to Wnt ligands.

### Phosphorylation-driven ubiquitination in receptor tyrosine kinase signaling

Many signaling pathways have inbuilt negative feedback loops to limit signaling after a stimulus has been applied excessively. Often, this includes targeted degradation of some part of the cascade, thus eliminating activated components and potentially limiting the sensitivity of the pathway to repeated stimulation. In the case of cell surface signaling molecules, such as receptor tyrosine kinases (RTKs), this elimination happens primarily through endocytosis after ligand stimulation^78^. Notably, in the case of RTKs, endocytosis does not always lead to degradation, however, but may also act to recycle receptors back to the plasma membrane. Ligand-induced endocytosis occurs for practically all RTKs, but its regulation is probably best understood for the epidermal growth factor receptor (EGFR).

EGFR can be endocytosed via multiple different mechanisms, but whether an endocytosed receptor is subsequently degraded or recycled, is mainly governed by phosphorylation-dependent ubiquitination^79^. Upon ligand stimulation, activated EGFR phosphorylates up to 9 tyrosines on its intracellular C-terminal tail, which acts as binding sites for various downstream signaling molecules, including the E3 ligase CBL^80^, which can subsequently ubiquitinate EGFR. It should be noted that the binding of CBL to phosphorylated EGFR is distinct from the binding of SCF adaptors to phospho-activated degrons (as described above). In fact, CBL binding—and subsequent ubiquitination—relies on the cooperative binding of EGFR through direct interactions between phosphotyrosines on the EGFR C-terminal tail and an SH2-like domain on CBL, as well as via the adapter growth factor receptor bound protein 2 (GRB2), which itself binds phosphotyrosines on EGFR via its SH2 domain^81^. Furthermore, the CBL ubiquitination site (i.e. secondary degron) is on the EGFR protein kinase domain and thus distal to the site of CBL/GRB2 recruitment^79^. Ubiquitination, which is mostly K63-linked, subsequently recruits reader enzymes, which facilitate the direction of EGFR to non-clathrin-mediated endocytosis and lysosomal degradation^79^ (Fig. 3). Unsurprisingly, given their outstanding mitogenic potential, deregulated RTKs (including EGFR) are major driving forces in many cancers. An example of deregulation involving the endocytic recycling/degradation machinery is CBL mutations found in myeloid neoplasms.

### PTM-regulated protein degradation in neurodegenerative diseases

Many neurodegenerative diseases, such as Alzheimer’s disease, Parkinson’s disease, Huntington’s disease (HD), and motor neuron disease, are associated with misfolded, often degradation-resistant proteins, which accumulate in neurons causing toxicity. One mechanism for the formation of these toxic proteins is the inclusion of poly-glutamine (polyQ) stretches to proteins, such as huntingtin (HTT), or androgen receptor (AR)^82^, making them aggregation-prone. Recent evidence has shown several examples, where the toxicity and stability of these proteins are affected by PTMs.

PolyQ-AR, for instance, which causes spinal and bulbar muscular atrophy (SBMA), is phosphorylated in an Akt-dependent manner at S215 and S792 upon stimulation with insulin-like growth factor 1 (IGF-1)^83^. Mechanistically this phosphorylation, which had previously been found to be required for degradative ubiquitination of AR by the E3 ligase MDM2 (ref. ^84^), appears to solubilize polyQ-AR from high-molecular-weight aggregates, allowing UPS-mediated degradation^83^. Conversely, polyQ-AR has also been suggested to be preferentially driven to aggregates by phosphorylation of another residue, S96, by CDK2, thus protecting it from degradation^85^. These sites constitute an interesting example of PTM-regulated degradation, which does not rely on the alteration of a degron per se, but rather on controlling a protein’s oligomericity/aggregation state (Fig. 3).

Similar events have been described in polyQ HTT, whose aggregation can be counteracted and clearance promoted by multiple phosphorylation events such as phosphorylation at S421 by Akt or inflammatory kinase IκB kinase (IKK)^31^, phosphorylation at T3 (ref. ^86^), and at S13 and S16 (refs. ^87, 88^). For S13 and S16 specifically, biophysical techniques could show suppression of membrane-induced aggregation and formation of β-amyloid fibrils in S13 and S16 phosphorylated HTT^89,90^. Interestingly, work in *C. elegans* has suggested that HTT acetylation at K444 could drive autophagic clearance of mutant HTT^91^. However, solid in vivo data of the relative physiological contributions of all of the currently known HTT PTMs is still outstanding, as disease progression is likely the sum of many regulatory layers and thus experimentally not straightforward to assess.

## Control of protein stability by acetylation

**Fig. 4:**
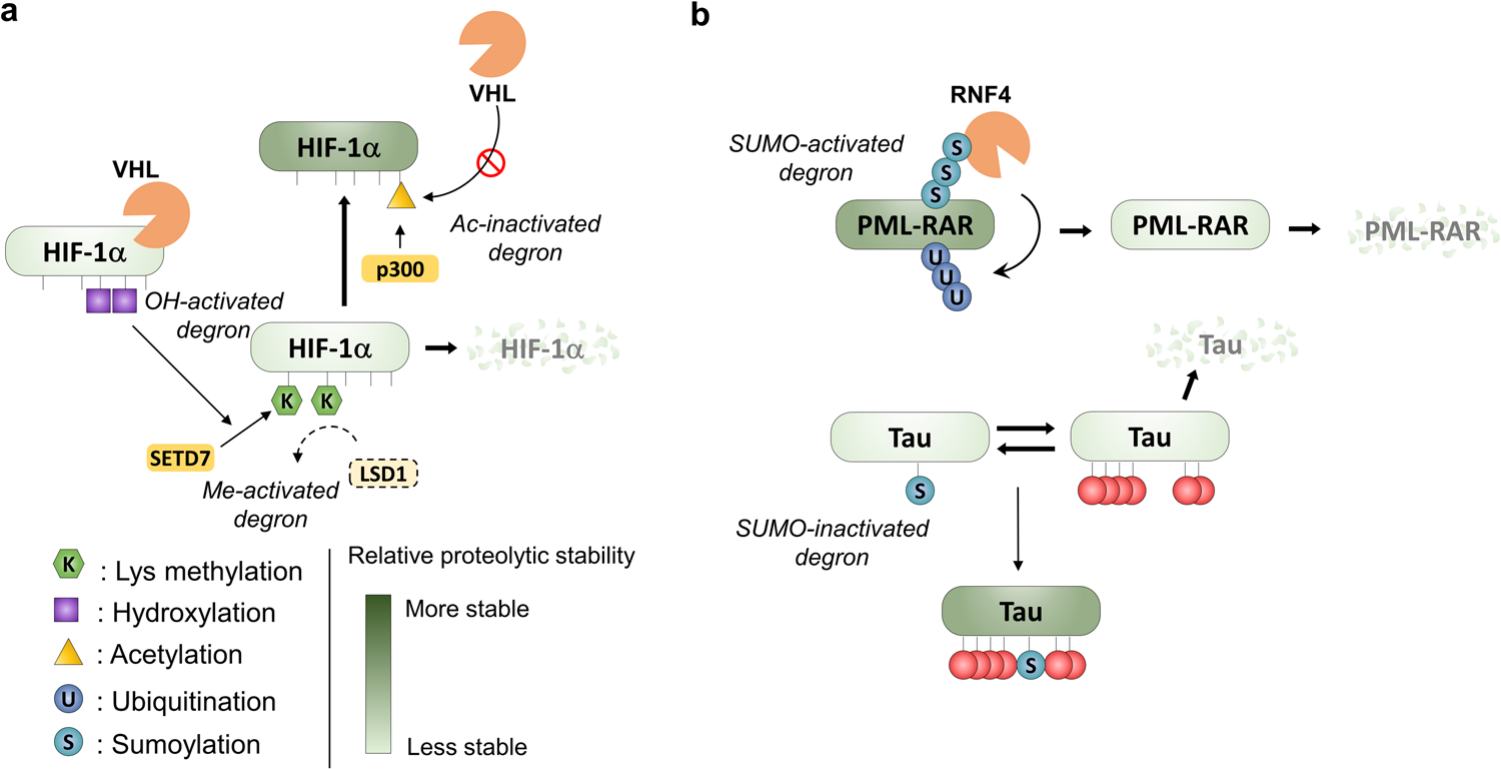
Protein stability controlled by methylation, acetylation, hydroxylation, and SUMOylation. **a** HIF-1α is regulated by acetyl-inactivated degron, hydroxyl-activated degron, and methyl-activated degron. p300 acetyltransferase generates acetyl-inactivated degron of HIF-1α and HIF-1α acetylation triggers stabilization in acetylation-dependent manner. Because hydroxylation stimulates methyl-activated degron of HIF-1α, hydroxylation reduces protein stability of HIF-1α directly or indirectly. **b** Examples of the regulation of protein stability by SUMOylation. SUMOylation of PML-RAR induces poly-ubiquitination and further degradation of PML-RAR. In contrast, SUMOylation and multiple phosphorylations of tau cooperatively increase protein stability of Tau.

The transfer of acetyl groups from acetyl-coenzyme A to the ε-amino acid groups of lysine residues results in charge neutralization, which can affect the properties of proteins much like other charge-changing PTMs, such as phosphorylation, altering protein conformation, function, protein-protein interactions, stability, and localization^92–94^. The most straightforward mechanism for acetylation-dependent protein stabilization is competition with ubiquitination when occurring on the same lysine residue. Indeed, acetylation-dependent protein stabilization of p53 occurs by preventing poly-ubiquitination by MDM2 (ref. ^95^), and acetylation of HIF-1α at K709 by the acetyltransferase p300, for instance, facilitates HIF-1α stabilization^96–98^, potentially through competition with ubiquitination at the same site^99^ (Fig. 4a).

## Control of protein stability by hydroxylation

The addition of hydroxyl groups to proline residues is a modification that is prevalent during protein secretion and cellular oxygen-sensing. Hydroxylation of proteins is catalyzed by 2-oxoglutarate-dependent dioxygenases and can take place on various amino acids, including proline, lysine, asparagine, aspartate, and histidine. The archetypal example of this PTM is HIF-α prolyl hydroxylation, which affects HIF-α protein stability via the VHL E3 ubiquitin ligase^100^. In the presence of oxygen, P402 and P564 are hydroxylated, leading to HIF-1α recognition by VHL and promotion of proteasomal degradation^101–103^. Lack of oxygen prohibits hydroxylation of HIF-1α followed by escaping of HIF-1α from the UPS leading to protein stabilization.

## Control of protein stability by glycosylation

Glycosylation of protein is an important biological modification that is crucial for various cellular functions, including protein stability, transcriptional regulation, signal transduction, and cell survival. Glycosylation influences cell surface receptors to modulate conformational change, protein turnover rate, and intermolecular interactions and subsequently changes the structure and functions of target proteins. For example, asparagine-linked (N-linked) deglycosylation of iron transporter ZIP14 at N102 was required for further proteasomal degradation^44^. This deglycosylation prior to ubiquitination-mediated degradation is regulated by cellular iron content and glycosylation-dependent inactivated degron provides insight into iron metabolism disorders. On the other hand, dysregulation in protein glycosylation could affect protein degradation and has been associated with the progression of diseases. The sugar side chains potentially stabilize a glycosylation-conjugated target protein, enhancing solubility, protecting it from proteolysis, and stabilizing intrachain interactions^104^. With the advantages of glycosylation engineering against target proteins, developing biological drugs targeting glycosylated proteins or glycan itself recently become a popular strategy in therapeutic antibody areas.

## Control of protein stability by sumoylation

Beyond the small chemical PTMs discussed above, the UPS and other degradation pathways can be regulated by polypeptide-PTMs, such as UBl modifiers, e.g., small ubiquitin-like modifier (SUMO), Neural precursor cell expressed, developmentally downregulated 8 (NEDD8), interferon-stimulated gene 15 (ISG15), ubiquitin-fold modifier 1 (UFM1), and ubiquitin-related modifier 1 (URM1)^105,106^. These modifications are added onto lysine residues, and can thus compete with other lysine-modifications such as ubiquitination, acetylation, and methylation^107,108^. Modification by UBls alters the interaction landscape of modified proteins, and as such, they can also alter a protein’s interactions with parts of the protein degradation machinery. For SUMO, this is most directly mediated by so-called SUMO-targeted ubiquitin ligases (StUbL), which specifically recognize SUMOylated substrates and ubiquitinate them^109^. A famous example is the degradation of the oncogenic fusion protein PML-RAR upon arsenic induction, which causes SUMOylation of PML-RAR, and subsequent recognition by the main mammalian StUbL, RNF4 (refs. ^110, 111^) (Fig. 4b). Also, indirect protein stability regulation by SUMO has been found. Indications for wide-spread cross-talk between SUMOylation and phosphorylation have been found^31,112^. Hyperphosphorylation and SUMOylation of the Alzheimer’s disease-associated protein tau, for example, have been shown to be mutually reinforcing leading to proteolytic stabilization of tau^113^ (Fig. 4b).

## Crosstalk between PTMs at degrons – Myc and P53

The number of experimentally observed sites for different PTMs greatly outnumbers the number of protein-encoding human genes^89^, and a recent proteomic study of lung cancer samples found that ~35% of phosphorylated proteins also carried at least one other methylation or acetylation site^114^. Consequently, individual PTMs on a given protein are being increasingly considered as part of regulatory PTM networks acting in combination to elicit functional outcomes^115^, including control of protein proteolytic stability. Molecularly characterizing PTM cross-talk is, however, challenging and larger PTM networks have only been elucidated for the most well-studied proteins. In the following, two such example cases are presented: MYC and p53.

### Regulation of MYC stability by multiple PTMs

**Fig. 5:**
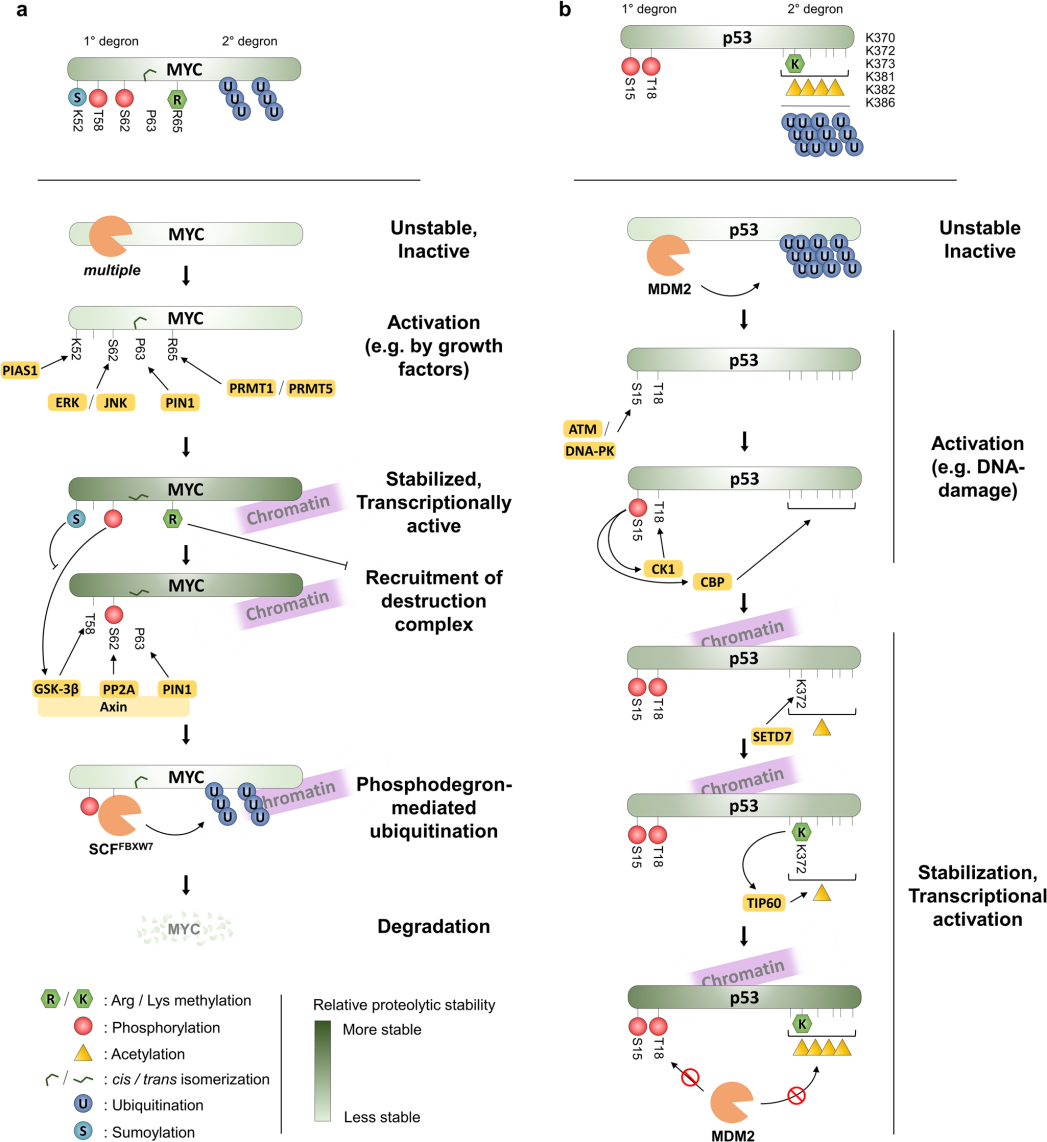
PTM cross-talk at degrons. **a** Examples of the regulation of MYC stability by different PTMs. In many cases, PTM networks are formed as one PTM acts as a priming factor for the next, by establishing protein-protein-interaction interfaces. **b** Examples of the regulation of p53 stability by PTMs. Note, multiple other PTMs have also been shown to affect p53 turnover in addition to the ones shown.

MYC proteins are a family of 3 master transcription factors: c-Myc (MYC), N-Myc (MYCN), and l-Myc (MYCL), with essential important roles in numerous biological processes, including cell proliferation, apoptosis, differentiation, and stem cell self-renewal, and they are overexpressed in many cancers^116^. As a promiscuous strong proto-oncogenic transcription factor, MYC protein levels are usually tightly regulated and numerous degradation pathways of MYC have been described, which ensure a short overall half-life for the MYC protein of ~20 minutes^117^. The most thoroughly elucidated degradation pathway hinges on two conserved phosphorylation sites: T58 and S62, and an elaborate interplay of kinases, phosphatases, prolyl isomerases, and E3 ubiquitin ligases uncovered by multiple decades of work in many laboratories. In short, stimulating signals cause MYC phosphorylation at S62 (e.g. by ERK) and *trans-cis*-prolyl-isomerization at P63 (by PIN1), which act together as an inactivated degron to stabilize and activate MYC. Subsequently, MYC destabilization and degradation are affected by phosphorylation at T58 (by GSK-3β), prolyl-isomerization at P63 back to *trans*, and dephosphorylation at S62 (by PP2A), thus creating a singly-phosphorylated (pT58) phospho-degron recognized by SCF^FBXW7^ (Fig. 5a).

This phospho-degron pathway has since been suggested to further be regulated by other PTMs. For instance, SUMOylation at K52 by the SUMO E3 ligase protein inhibitor of activated STAT 1 (PIAS1), was suggested to lead to upregulation of S62 phosphorylation by c-Jun N-terminal protein kinase 1 (JNK1). Simultaneously, PIAS1 suppresses phosphorylation of T58, thus preventing the formation of the T58 phospho-activated degron^118,119^. In this case, however, additional mechanisms are likely to be involved, since destabilization of MYC mutated at K52 is not fully dependent on FBXW7 (Ref. ^120^). MYC arginine methylation by PRMT1 and/or PRMT5 has also been suggested to alter MYC stability through the T58 phospho-degron pathway, with methylation at R65 potentially suppressing T58, thus stabilizing MYC^121^.

MYC degradation has also been linked to numerous other E3 ligases and pathways, some of which are dependent on other PTMs. For example, MYC has been shown to be a substrate of the acetyltransferases cAMP response element-binding protein (CREB) binding protein (CBP), p300, GCN5, PCAF, and Tat-interactive protein 60 (TIP60)^122^. Acetylation by p300, GCN5, and TIP60 has been reported to at least partially stabilize MYC by suppressing UPS-mediated degradation^122,123^. In line with this finding, SIRT1 has been shown to directly remove acetylation of MYC, resulting in decreased protein stability of MYC^124^. The exact details of the effects of MYC acetylation on its stability are, however, still not well understood. For instance,MYC has been shown to be hyperacetylated and destabilized upon HDAC inhibitor treatment^125^.

### Examples in the regulation of p53 stability

The tumor suppressor p53 is mutated in approximately 50% of human malignancies and plays a crucial role in tumor suppression in response to genotoxic stress^15^. Vast amounts of research have been invested into p53, and consequently, it ranks amongst the top proteins with the most annotated sites of different PTMs^115^, with over 300 mapped PTM sites^126^. Given the breadth, depth, and complexity of the knowledge on p53 regulation by PTMs amassed over the last four decades, only a few examples thereof shall be presented here, and the interested reader is encouraged to delve into excellent recent reviews on the subject^126^.

p53 is a generally short-lived protein, and activation of it is usually accompanied by stabilization of the protein. Over 30 different E3 ligases have been reported to ubiquitinate p53 with varying effects on its stability^127^. Most classically, bulk turnover of unmodified p53 is attributed to a single E3 ligase, MDM2, which binds the N-terminus of p53 at residues 18–28 (ref. ^128^) and ubiquitinates it on six C-terminal lysines: K370, K372, K373, K381, K382, and K386, thus shuttling the protein to the UPS. Consequently, much of the regulation of p53 stability hinges on these two parts of the p53 degron: the primary degron at residues 18–28; and the secondary degron at 370–386. For example, after DNA damage, the first degron is inactivated by sequential phosphorylation: first of S15, which allows for phosphorylation of T18 by, e.g., casein kinase 1 (CSNK1A1/CK1), thus lowering the affinity of p53 for MDM2 (ref. ^129^). On the other hand, inactivation of the secondary degron by acetylation (thus making the lysine residues incapable of acting as ubiquitin acceptors) will lead to the stabilization of p53 (ref. ^95^). There is also evidence showing crosstalk between N-terminal phosphorylation of p53 at S15 among other sites, and activation of acetylation of the C-terminal lysines by increased recruitment of the acetyltransferase CBP, thus strengthening the stabilization of p53 (refs. ^130, 131^) (Fig. 5b).

In addition to acetylation, lysine methylation has also been shown to affect p53 stability and activity, even if the situation here is more complex. For instance, K370 and K382 di-methylation were shown to promote p53 activity, probably due to the recruitment of p53 binding protein 1 (TP53BP1) via its Tudor domain^132^, thus potentially sterically occluding the p53 secondary degron preventing ubiquitination. In contrast, mono-methylation of K370 and K382 has been shown to suppress p53 activity by recruiting transcriptional repressors^133,134^.

Analogously to the pS15-mediated recruitment of CBP above, mono- and di-methylation of K372 by SETD7 has been proposed to increase the stability of chromatin-bound p53 (ref. ^135^), probably by increasing recruitment of the acetyltransferase TIP60, which in turn acetylates the other secondary degron lysines preventing ubiquitination^136^. In the overall context of p53 regulation, the contribution of SETD7-dependent K372 methylation is, however, still unclear with different murine genetic models providing conflicting evidence of its effects on p53 abundance and transcriptional activity^136,137^, as well as data from cellular models showing K372 methylation-independent p53 activation by SETD7 (ref. ^97^). Numerous other PTMs have also been reported to affect p53 stability and activity. As for the case of K372 methylation, however, many of them have later been questioned or contradicted––especially with regard to their physiological relevance. Consequently, a clear picture of the underlying molecular mechanisms has proven tricky to elucidate, despite considerable efforts.

## Applying protein stability proteomics for the study of PTMs

All of the examples presented above are the result of years—or sometimes decades—of meticulous biochemical experimentation. Many were found individually and serendipitously, and the number of proteins with PTM-controlled degrons is likely to be far greater than currently known. Just out of the estimated ~65% of the proteome turned over by the UPS^44^, many proteins are likely controlled by additional PTMs, many of which on the same polypeptide, or even immediately proximal to the ubiquitination site^14^.

Currently, PTMs detected by mass spectrometric techniques far outnumber the number of functionally understood PTMs. In the case of phosphorylation, for instance, functional information exists for fewer than 3% of the mapped human protein phosphosites^138^. Indeed, a major challenge of modern proteomics lies in shedding light on this “dark” phosphoproteome. Several complementary approaches exist for this, such as predicting functionality using pre-existing multiomics data^139^ or screening libraries of phospho-deficient mutants across different conditions^140^. Another promising, more directed approach is to use functional proteomics to systematically assess the effects of PTMs on the properties of proteins.

### System-wide investigation of PTM effects on proteolytic stability

Recent developments in mass spectrometry-based proteomics make it possible to monitor changes in both proteolytic^54,141–143^ and thermal stability^144^ of proteins under steady-state conditions or following perturbations. Protein turnover rates can be measured using dynamic^141,145^ or pulsed stable isotope labeling with amino acids in cell culture (SILAC)^146^. The keystone of these technologies is the ability of mass spectrometers to distinguish peptides with identical sequence, but carrying light or heavy isotope-labeled amino acids—most commonly arginine and lysine are used—and quantify their relative abundances. Following a switch from a light amino acid to a heavy amino acid-containing cell medium, samples are collected at several time points. For any given protein, the rate of heavy amino acid incorporation after the medium switch will be proportional to its synthesis rate, while corresponding light amino acid-containing peptides will decrease in abundance proportionally to its degradation rate. Thus, quantitative data from these experiments can be used to determine protein turnover rates.

These approaches can also be extended to monitor turnover rates of specific modified peptides by combining dynamic or pulsed SILAC with enrichment strategies for different types of PTMs^147,148^. Such a strategy can provide clues to which PTMs potentially have an impact on the proteolytic stability of a protein. Overall, stabilizing PTMs would be expected to show significantly slower turnover rates than the unmodified protein, while the opposite would be true for destabilizing PTMs. Caution needs to be taken, however, as different apparent turnover rates of PTM-modified versions of a given protein can result from mechanistically unrelated sources. These could include, for instance, differentially modified subpopulation of protein which has distinct subcellular localization and thus underlies different proteolytic control^84,149^. Similarly, cases in which PTMs accumulate onto a protein over time can result in skewed apparent turnover rates for modified and unmodified versions, even if the modification itself is not affecting protein stability per se. This method does, however, enable rapid short-listing of PTM sites potentially affecting a protein’s proteolytic stability, thus limiting the search-space considerably.

### Perturbation-based investigation of PTM effects on proteolytic stability

**Fig. 6:**
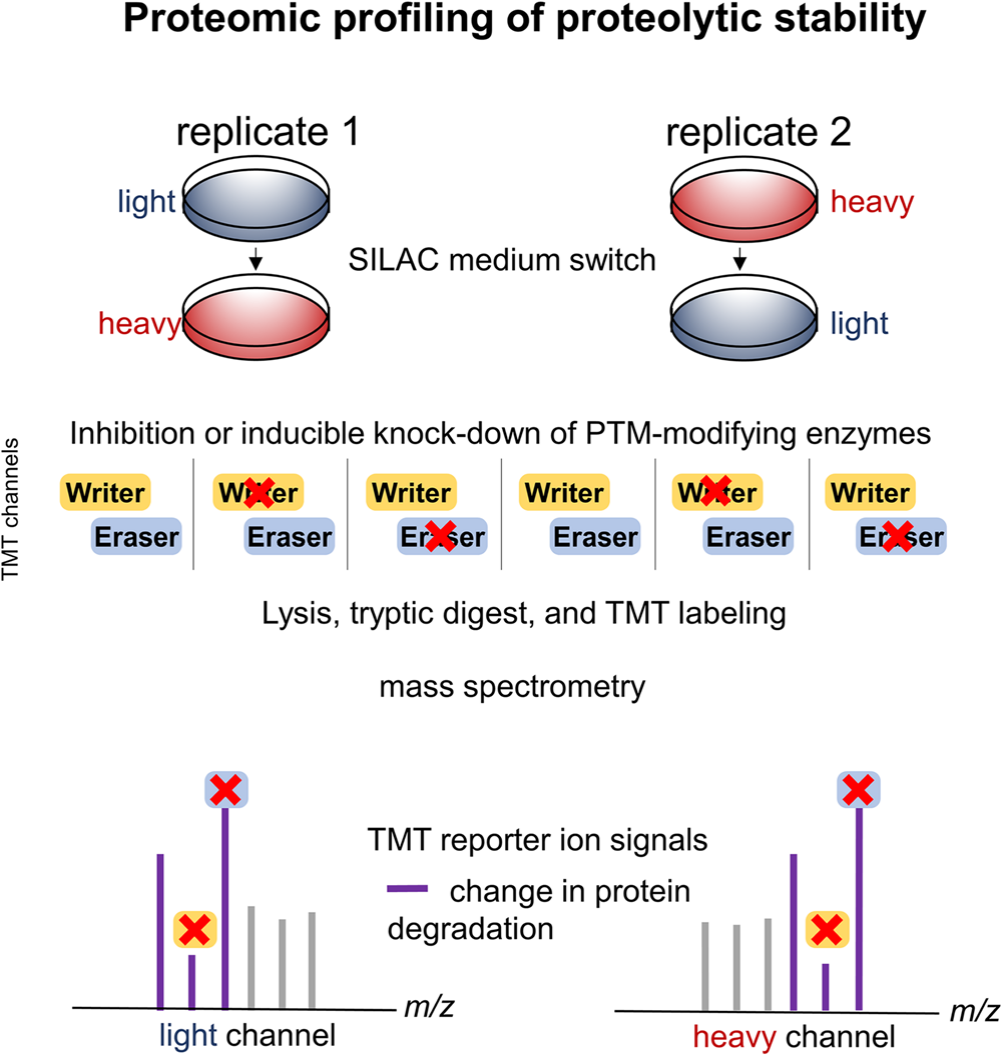
Proteomic profiling of effects of PTM modulating enzymes on proteolytic stability. Schematic for using multiplexed proteome dynamics profiling for detecting the effects of PTM writer and eraser enzymes on protein degradation. Cells are cultured in light SILAC medium or heavy SILAC medium. Following a switch to heavy SILAC medium (replicate 1) or light SILAC medium (replicate 2), and subsequent inhibition of writer or eraser enzymes, samples are collected after a suitable time point, lysed, digested with trypsin, labeled with isobaric mass tag reagents (TMT), and pooled. Next, samples are processed by mass spectrometry. Tandem mass spectra generated from light and heavy SILAC signals will contain TMT reporter ions that can be used for relative quantification of changes in degradation rates between the different conditions (control, inhibition of writer enzyme, inhibition of reader enzyme) from the two biological replicates.

An alternative, complementary strategy for assessing the effect of PTMs on protein stability would entail interfering with the function of the PTM-placing enzyme (writer enzyme) and/or the enzyme responsible for the removal of the PTM (eraser enzyme) (Fig. 6). Recently, it has become possible to systematically measure changes in protein degradation following cellular perturbations by combining dynamic SILAC with multiplexed quantitative mass spectrometry using isobaric mass tag labeling^150^. Switching the cell medium from light to heavy amino acids shortly prior to the perturbation event—such as drug treatment—enables separately resolving effects of perturbations on synthesis and degradation to the subsequent abundance changes of proteins. This technology, termed multiplexed proteome dynamics profiling (mPDP), makes it possible to precisely assess and compare to each other the effects of multiple perturbations. Currently, up to sixteen different conditions can be assessed in one experiment^151^, on protein degradation and synthesis rates. A PTM-centric experimental strategy could thus entail performing mPDP experiments where a selection of relevant writer and eraser enzymes are inhibited using either tool compounds or inducible knockdown strategies (Fig. 6)^152^. This will reveal which proteins are proteolytically stabilized or destabilized under different conditions. Specific PTM enrichment experiments can then be performed to investigate, which PTMs change in their abundance following the inhibition of specific enzymes, and to link that information to the changes in protein proteolytic stability. Subsequently, mutations of specific PTM sites could be performed by generating gene-edited cells to validate findings in a follow-up mPDP experiment.

## Perspectives and implications for the development of future therapeutics

The large variety of molecular mechanisms underpinning protein stability regulation by PTMs highlights the network nature of much of cellular signaling. Given the relatively recent descriptions of methyl-activated degrons and -inactivated degrons, it remains to be seen, whether the molecular mechanisms behind these control elements follow the same trends as seen with the more thoroughly characterized phospho-activated and –inactivated degrons. Furthermore, additional mechanisms of regulation are constantly being discovered, such as sequestering of E3 ligases and substrates by phase separation^153^, which is also controllable by PTMs^154^.

The increasing knowledge about the additional layers of protein stability regulation has straightforward implications for the development of new therapeutics. Many prominent oncogenes such as transcription factors, often include large intrinsically disordered regions, lack enzymatic activity and defined binding pockets, and thus have sometimes been deemed “undruggable” by traditional small-molecule approaches^155^. Crucially, the PTM networks controlling the protein stability and abundance of many of these factors consist of writer and eraser enzymes, which may provide more readily “druggable” targets^156^. For instance, the prominent oncogene MYC relies on dephosphorylation of its phospho-inactivated degron S62 by PP2A for targeting the protein for degradation. Consequently, activating PP2A by inhibiting its inhibitors or directly activating it with small molecules are interesting candidates for indirect approaches in targeting MYC-reliant cancers^157^.

Protein-targeting chimeric molecule (PROTAC) is an exciting strategy for inducing targeted protein degradation^158^. The first proteolysis-targeting chimera PROTAC consists of a doubly phosphorylated peptide derived from IκBα and recruits β-TrCP E3 ligase—which covalently binds to its substrates via linker^159^—and an inhibitor of methionine aminopeptidase-2 (MetAP-2), ovalicin. The chimera could then simultaneously bind to MatAP-2 and recruit β-TrCP E3 which led to the degradation of MatAP-2. This study showed that PTM-regulated degron sequences could be harnessed to induce protein degradation using PROTACs. PROTACs have since developed into a subfield of their own and interested readers are encouraged to delve into recent specialized reviews on the subject^160^.

Currently, the complexity of PTM-driven regulation of protein stability is largely a function of how much research attention a protein has received, as exemplified by the cases of p53 and MYC discussed above. Given the pervasive nature of this form of control of protein abundance, we speculate that a large proportion of the regulatory landscape is yet to be discovered. Encouragingly, the expansion of knowledge of this landscape is providing an increasing number of accessible drug targets, including in the recently characterized methyl-activated degrons^29,161^.

## Conclusions

The occurrence of diverse PTMs regulating protein stability, acting sequentially and/or in concert, is crucial to initiate, terminate, or fine-tune the outcome of signaling pathways in human diseases. The diverse types of PTMs and signaling cascades that lead to protein degradation or stabilization show how a complex circuitry of PTMs is generated and erased dynamically to regulate the function of disease-related proteins^162^. PTMs act by reversibly altering a protein’s physicochemical properties, thus changing conformation, function, and/or interaction interfaces. In the context of known examples of PTM-regulation of protein stability, these changes often act by bridging the space between an E3 ligase and its substrates.

Numerous PTMs have been identified to date acting on a subset of amino acid side chains. Notably, especially lysine residues with their chemically versatile primary amine group are subject to a variety of PTMs, including methylation, acetylation, SUMOylation, and ubiquitination. These different types of modifications provide mutually exclusive, alternative properties to the side chain, thus essentially creating miniature switches or carriers of transient information in the form of different chemical moieties. Direct competition between lysine acetylation and ubiquitination, for instance, has been suggested to be a critical modulatory mechanism for inhibiting protein ubiquitination, as in the case of p53, p73, NF-E4, Runx3, Smad7, SREBP1a, and SREBP2^17,84,163–166^.

The field of PTM-regulation of protein stability is mostly still in a state of a growing accumulation of single example cases found using traditional biochemical and molecular biological approaches. Similarly, significant challenges for future research will be the elucidation of writer and eraser enzymes, as well as the assessment of physiological and pathological functions of the different PTMs. Furthermore, identification and molecular understanding of E3 ubiquitin ligase specificity are sorely lagging.

We believe, however, that the application of novel proteomic techniques offers the promise of vastly accelerating the process of identifying and elucidating the function of PTM-controlled protein stability modules. This expansion of our knowledge will be needed not only to identify novel drug targets in this domain of protein regulation but also to shed light on the generalizable trends, including potential sequence motifs, specificity signatures, etc., underlying the richness of molecular mechanisms already known today.
